# A Case of Doxycyclin-induced Photo-onycholysis with Dermoscopic Features

**DOI:** 10.4274/balkanmedj.galenos.2019.2019.11.22

**Published:** 2020-02-28

**Authors:** Ömer Faruk Elmas, Necmettin Akdeniz

**Affiliations:** 1 Department of Dermatology, Ahi Evran University School of Medicine, Kırşehir, Turkey; 2 Department of Dermatology, İstanbul Medeniyet University School of Medicine, İstanbul, Turkey

A 16-year-old boy was referred to us with asymptomatic nail discoloration affecting his fingernails. The patient was receiving treatment with 200 mg/day oral doxycycline administration for six weeks due to brucellosis. His remaining medical history was unremarkable except for intense sun exposure. The dermatological examination revealed a distal black and brown discoloration with half-moon shaped edges affecting all the fingernails (Figure 1). On dermoscopic examination, bluish black discoloration, brown dots, and proximal brown discoloration with sharp linear edges were observed (Figure 1). No yellow oil spots, blood spots, pitting, or subungual hyperkeratosis were detected. No cutaneous lesions were observed elsewhere. Direct microscopic examination with potassium hydroxide preparation did not show the presence of fungal element. Bacteriological investigations revealed no infectious agents. A diagnosis of doxycycline-induced photo-onycholysis was made.

The patient was referred to the infectious diseases department, and doxycycline administration was stopped. The patients was initiated on a combination of rifampicin and trimethoprim-sulfamethoxazole administration. A control examination was scheduled for six weeks later, but the patient did not present again. Written informed consent was obtained from the patient.

Onycholysis is the separation of the nail plate from the nail bed and may involve one or more nails. The separated part of the nail often shows a half-moon shape and appears whitish due to the separation of the nail plate from the vascular nail bed. Some cases of onycholysis may show a bluish black discoloration due to the subungual accumulation of dirt (1). Our patient also showed a bluish black discoloration, which was possibly caused by exogenous dirt.

Physical trauma and some dermatological and general medical conditions may cause onycholysis. Tetracycline, ciprofloxacin, griseofulvin, and docetaxel were reported to cause onycholysis (1,2,3).

Photo-onycholysis is a rare phototoxic reaction caused by prolonged and intense ultraviolet exposure that results in onycholysis. It has been suggested that doxycycline-induced phototoxicity is associated with lumidoxycycline, which is a photo product of doxycycline (3,4). In our case, the brown nail discoloration with sharp linear edges and the history of intense sun exposure favored a diagnosis of photo-onycholysis. Although the evidence for photo-onycholysis was indirect, the sparing of the photo-protected toenails strongly supported the diagnosis.

Dermoscopic findings of many nail conditions have been described in detail. However, to the best of our knowledge, dermoscopic features of photo-onycholysis have not been identified previously. In the present case, the dermoscopic examination facilitated the diagnosis by enhancing the appearance of the brown dots and proximal brown discoloration with sharp linear edges, which are suggestive of photo-onycholysis.

## Figures and Tables

**Figure 1:**
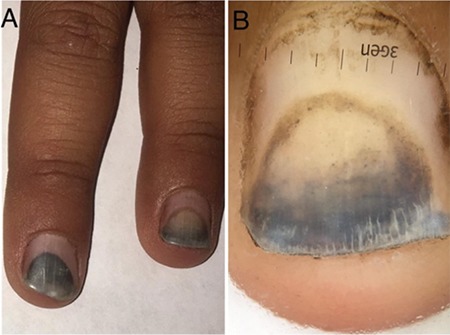
(a) Distal bluish black nail discoloration with a half-moon shape affecting the fingernails, (b) Dermoscopic examination shows a bluish black discoloration, brown dots, and proximal brown discoloration with sharp linear edges.
